# Nanoscience in Action: Unveiling Emerging Trends in Materials and Applications

**DOI:** 10.1021/acsomega.4c10929

**Published:** 2025-02-17

**Authors:** Kevin J. Hughes, Magesh Ganesan, Rumiana Tenchov, Kavita A. Iyer, Krittika Ralhan, Leilani Lotti Diaz, Robert E. Bird, Julian Ivanov, Qiongqiong Angela Zhou

**Affiliations:** † CAS, a Division of the American Chemical Society, Columbus, Ohio 43210, United States; ‡ ACS International India Pvt. Ltd., Pune 411044, India

## Abstract

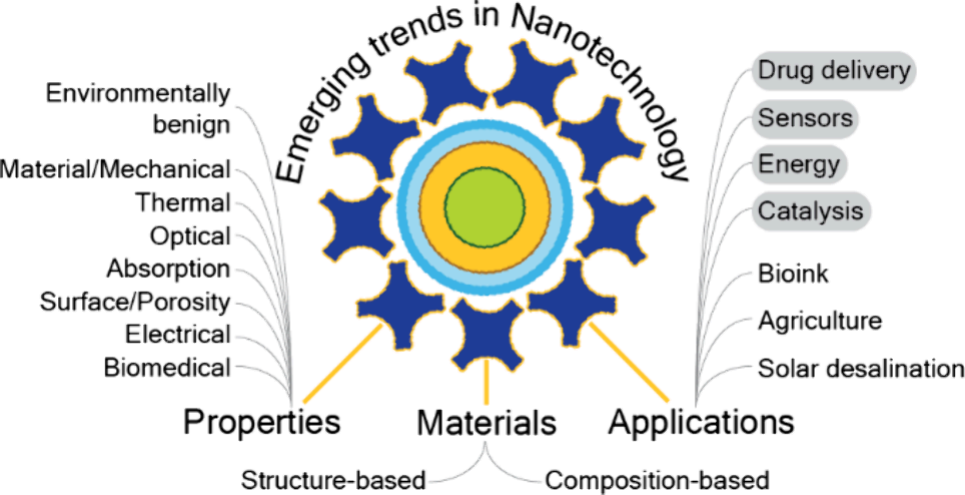

Since their inception in the early 1960s, the use of nanoscale materials has progressed in leaps and bounds, and their role in diverse fields ranging from human health to energy is undeniable. In this report, we utilize the CAS Content Collection, a vast repository of scientific information extracted from journals and patent publications, to identify emerging topics in this field. This involves understanding trends, such as the growth of certain topics over time, as well as establishing relationships among emerging topics. We achieved this by using a host of strategies including a quantitative natural language processing (NLP) approach to identify over 270 emerging topics and subtopics across three major categories—materials, applications, and properties—by surveying over 3 million documents spanning across two decades in the nanoscience landscape. This wealth of information has been condensed into several conceptual Trendscape maps and other data visualizations, providing metrics related to the growth of identified emerging concepts, and grouped into hierarchical classes, and the connections between them have been explored. Our extensive analysis taking advantage of an NLP-based approach along with robust CAS indexing provides valuable insights in the field that we hope can help to inform and drive future research efforts. In a series of interconnected papers, we will present our findings from this project, with a focus on four major applications of nanoscale materials—drug delivery, sensors, energy, and catalysis—to provide a more comprehensive and detailed picture of the use of nanotechnology in these fields.

## Introduction

Nanoscience, the study of materials and phenomena on the nanometer scale, has emerged as a frontier discipline at the intersection of physics, chemistry, biology, and engineering, with profound implications for various industries and scientific disciplines.

Nanomaterials have dimensions on the scale of one billionth of a meter. Specifically, IUPAC has defined nanoparticles as those having sizes in the range of 1–100 nm.^1^ Materials having at least one dimension, pores, film thickness, or surface features in the 1–100 nm range are also considered to be nanomaterials.^2,3^ At the nanoscale, materials exhibit unique properties and behaviors that are distinct from their bulk counterparts, enabling innovative applications in areas such as electronics, medicine, energy, and environmental remediation.

The concept of nanotechnology emerged for the first time in the lecture “There’s plenty of room at the bottom” delivered by the physicist and Nobel Prize laureate Richard Feynman at the American Physical Society meeting in 1959.^4^ This presentation is considered to be a seminal event in the history of nanoscience, as it inspired the conceptual beginnings of the field decades later.^5^ In this lecture, Feynman aimed to draw the attention of the audience to the advancement of technologies to produce ultrasmall objects built of very few atoms or molecules. In the 1990s, with the rapid rise of the nanotechnologies, scientists recalled Feynman’s lecture and found that much of what they were contemplating was actually envisaged 30–40 years earlier. Since then, Richard Feynman has been considered the founder of this new area of science and technology. The interest in exploring the size-dependent properties and utilizing these properties to enhance or create applications led to rapid growth in the number of publications related to nanomaterials beginning in the 1990s. The initial years of nanoscale research were focused on synthesis methods and morphology. In recent years, the relentless pursuit of understanding and harnessing nanoscale phenomena has become a focal point for researchers worldwide. It has now reached a point where products using nanotechnology are commercially available.^6,7^

The avalanche progress of nanotechnologies by the end of the 20th century is associated with the fundamentally novel methods of visualization and characterization of nanoobjects at atomic resolution developed in the 1980s.^8^ In 1986, one-half of the Nobel Prize in Physics was awarded to Ernst Ruska “for his fundamental work in electron optics, and for the design of the first electron microscope”, and the other half was jointly awarded to Gerd Binning and Heinrich Rohrer “for their design of the scanning tunneling microscope”.^9^ The atomic force microscope (AFM), created in 1986 by Binnig, Quate, and Gerber,^10^ is currently one of the most commonly used methods for the investigation of nanostructures.^11−15^ It finds numerous applications in a number of areas of natural sciences such as solid-state physics, semiconductor physics, physics and chemistry of polymers, surface physics, molecular and cell biology, and medicine.^16^ As scientists started building more devices to facilitate the magnification and visualization of the particles that are only a few nanometers in size, new breakthroughs in biology also paralleled these, such as advanced studies of DNA.^15^

The most recent celebration of the advances in nanoscience came with the Nobel Prize in 2023. The Nobel Prize in Chemistry was awarded to Bawendi, Brus, and Yekimov “for the discovery and development of quantum dots”,^17,18^ semiconductor nanoparticles with optical and electronic properties determined via quantum mechanical effects. Quantum dots are already used in multiple scientific areas, from physics and chemistry to medicine.^19−24^ The 2023 Nobel Prize in Physiology or Medicine awarded to Karikó and Weissman “for discoveries on nucleotide base modifications that led to the development of effective mRNA vaccines against COVID-19”^25^ also has its nanoscience attribute—lipid nanoparticles used as a delivery vector for the vaccine.^26^

By synthesizing, manipulating, or combining matter at the nanoscale, researchers can tailor properties, such as conductivity, catalytic activity, and optical properties, to meet specific needs. Additionally, the ability to precisely control size, shape, and composition opens doors to unprecedented functionalities, paving the way for transformative technologies.^27^ Some of the tunable properties are the melting point, band gap, fluorescence, electrical conductivity, magnetic permeability, surface area, and chemical reactivity.^28^

The high surface to volume ratio of nanomaterials can significantly enhance surface-related properties such as catalytic activity,^29,30^ charge storage capacity,^31^ ion storage capacity,^32^ and gas storage capacity, which are important in multiple applications including energy conversion and storage^33^ and environmental remediation.^34^ Their small size and ability to customize nanomaterials have made them valuable in life sciences applications such as drug delivery^26,35,36^ and diagnostics.^37−39^ Nanoporous catalysts^40,41^ and membranes^42,43^ help achieve high product selectivity and precise gas separation,^44^ respectively. Due to their distinct properties, nanomaterials can be highly responsive to environmental variations, leading to their use in different types of sensors.^45−47^ Due to their high strength as well as low density relative to conventional materials, nanomaterials and their composites are preferred in engineering applications.^48−50^

Research in nanoscience expanded very rapidly in the past few decades, resulting in an enormous number of publications. In this work, we aim to summarize this huge volume of publications to get a broader picture of the nanoscience research landscape using data from the CAS Content Collection. We particularly focused on the emerging applications of nanoscience research, providing a futuristic perspective of this important research area.

Using data from the CAS Content Collection,^51^ the world’s largest human expert-curated collection of scientific data, we present a landscape view of the current research in the field of nanoscience. Querying this database resulted in identifying roughly 3 million journal and patent documents in the field of nanoscience published since 2003. Using natural language processing analysis^52^ focused on recently published documents, we identified emerging and prevalent topics and clustered/grouped them into categories such as applications, materials, and properties. Since the use of artificial intelligence (AI) continues to proliferate across diverse fields, we chose to dedicate a small section to analyzing the role of AI in nanoscience, which remains very much in its infancy/early stages but has a promising future.

In addition to this paper and considering the vastness of the field and the abundant data available, we have chosen to narrow our focus and probe in greater detail four topics related to applications of nanoscience and nanotechnology, namely, drug delivery, sensors, catalysis, and energy-related applications. These four topics are chosen for detailed analysis as they ranked high in terms of the number of publications within the nanoscience data set. The results of these analyses are presented in separate articles.

## Nanotechnology: Emerging Topics and Trend Landscape

In this section, we identify and describe the most active areas of research and development within the broad landscape of nanoscale materials (nanotechnology). Guided by quantitative journal and patent document data analysis, we built a conceptual Trendscape showing a hierarchy of active and emerging research concepts. We selected key features (scientific concepts) in the Trendscape and cited examples of the most influential research that is driving their emergence with recent literature examples. Finally, additional quantitative analysis was used to explore the connections between scientific concepts in the Trendscape.

**Figure 1:**
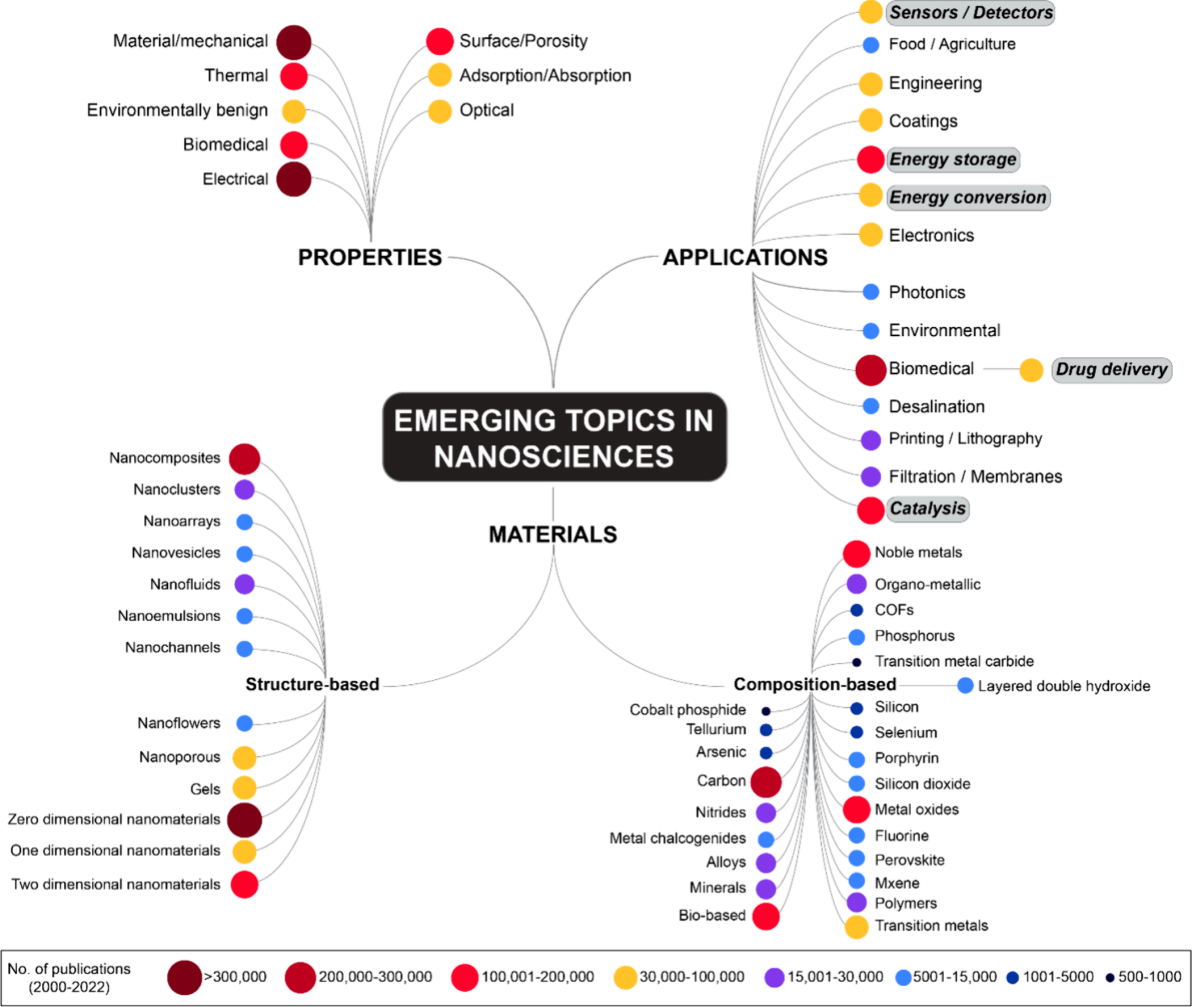
Trendscape map of the applications, materials, and properties in nanoscience that showed high growth in recent years.

**Figure 2:**
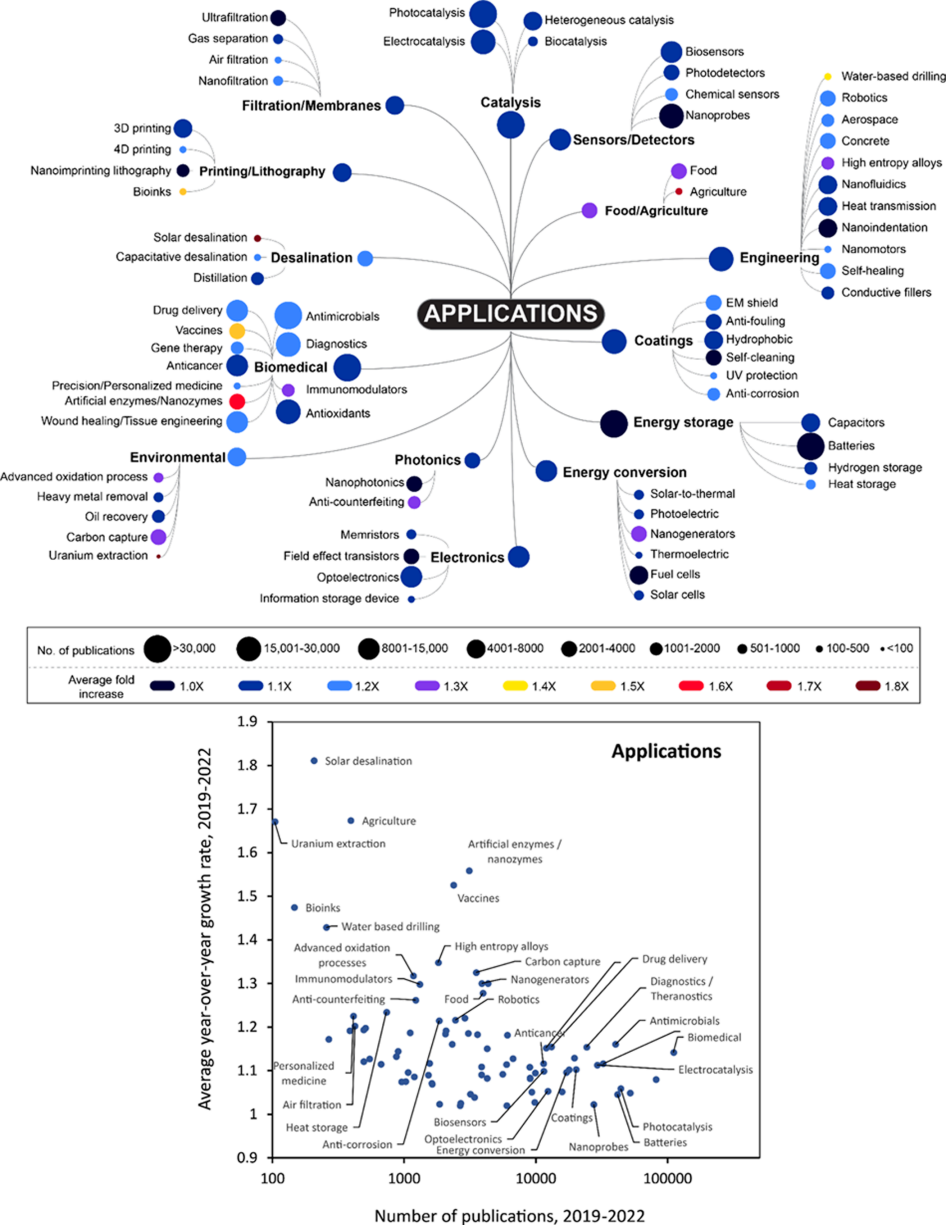
(Top) Conceptual Trendscape map and (bottom) average 2019–2022 growth rate versus number of publications over the time period that references applications involving nanoscale materials.

Figures 1, 2, 4, and 6 are conceptual Trendscape maps of emerging topics in nanoscience. These were made by first identifying journal and patent documents containing nanoscale material topics in the CAS Content Collection published since 2019. This resulted in a set of over 1.3 million documents. NLP analysis,^52^ guided by subject matter experts, was then used to extract and group scientific concepts from these documents (outlined in the Supporting Information). A combination of publication frequency, growth rate, and size of the field since 2019 was utilized to narrow down emerging topics showcased using a combination of colors and symbols in the maps. Identified concepts were arranged in a hierarchical manner, effectively dividing concepts among the broader branches of applications, materials, and properties. Figure 1 is a combined map providing a zoomed-out view of the major branches (application, properties, and materials) and is limited to the first level of branching. It is important to note that the ends of the branches in the map and the individual data points in the graphs represent groups of terms summarized by the label. For example, the term “food” includes concepts such as food safety, food processing, food additives, and food preservation. Similarly, terms with alternate forms such as nanoenzyme, nano-enzyme, and nanozyme, as well as singular and plural forms, have also been grouped.

Of the three sections in Figure 1, a detailed hierarchy of concepts in applications, materials, and properties is presented in Figures 2 (top), 4 (top), and 6, respectively. In the applications section of Figure 1, we have highlighted (using gray boxes) applications with a large corpus of documents that were subsequently pursued for in-depth analysis and include both biomedical applications such as drug delivery and nanosensors and nonbiomedical applications such as energy storage and conversion and catalysts. In the applications section of the map, the most prominent emerging branches are biomedical (driven largely by diagnostics, antimicrobials, antioxidant, and drug delivery), catalysis (electro- and photocatalysis), and energy storage. Driven by the need to achieve better healthcare and control global warming, considerable research attention is focused on biomedicine, sustainable energy, and environmental remediation. The major applications involving nanoscience reflect this trend, highlighting the efforts to leverage the advancements in nanoscience to achieve the desired results in these in-focus research areas. In the Materials section, the most active areas of research include zero-dimensional (0D) and two-dimensional nanomaterials (2D), carbon-based materials, naturally derived materials including cellulose and lipids, and noble metals. Nanoparticles, which are 0D, are the most commonly synthesized type dimension of nanomaterial, followed by 2D, which can be attributed to very high research interest in materials such as graphene.^53^ The major contributors to carbon-based materials are graphene and carbon nanotubes. Besides these, other applications of nanoscale materials with a relatively smaller corpus of journal and patent documents include environmental remediation, desalination, and the food and agricultural industry.

In terms of properties, the most abundant ones with the highest degree of prevalence in these documents are related to the major applications. Surface properties and porosity are important for catalytic, filtration/membrane, and sensing applications Nanomaterials are widely used in these applications as nanomaterials significantly enhance these properties due to their high surface area compared to the bulk materials. On the other hand, applications such as sensors, energy storage and conversion, and photonics depend heavily on the electrical and electronic properties of the materials.^54^ Other prevalent properties include biomedical, mechanical, thermal, adsorption, and optical properties. Adsorption of the reactant molecules on the catalyst’s surface is an important step in catalytic reactions, due to which it is one of the widely studied properties.^29^

The bottom panels of both Figures 2 and 4 compare the total number of publications against the average year-over-year growth rate of these publications from 2019 to 2022, further deepening our understanding of the emerging concepts in the data. Concepts that are referenced in a relatively low number of publications but with a high growth rate are found in the upper left section of the graphs, while more mature concepts with a relatively lower growth rate are found in the lower right section. The former might be concepts that are starting to emerge and would be worth tracking in the future, while the latter are concepts that are relatively well-established. Solar desalination, agriculture, uranium extraction, and agriculture are some of the applications that have a low number of publications and high growth rates. Applications such as artificial enzymes/nanozymes, vaccines, high entropy alloys, and carbon capture possess both high publication numbers and high growth rates. Artificial enzymes and nanozymes possess similar catalytic activities as natural enzymes, without the drawbacks of natural enzymes such as high cost and low stability.^55,56^ Research on carbon capture has been pursued due to the urgency to achieve net-zero emissions by the year 2050 and the need for the decarbonization of industries.^57,58^ Nanoporous materials such as metal organic frameworks, zeolites, and activated carbon are promising candidates for carbon capture.^59^

## Applications

In the section below, we will highlight concepts within the Trendscape map of especially high growth from 2020 to 2023.

Carbon capture nestled under environmental remediation is an example of an application showing moderate growth in recent years, with publications increasing on average 1.3-fold every year since 2020 (Figure 2 (top)). Interest in decreasing the levels of carbon dioxide in the atmosphere by capturing and storing it before release from industrial exhausts is on the rise, driven largely by accelerated global warming. The use of nanomaterials for this purpose has grown in recent years since surface area and porosity play important roles in this process. More than 90% of the studies associated with use of nanomaterials in carbon capture revolve around their use as catalysts for the reduction of CO_2_ to useful and relatively harmless chemicals or the capture and storage of CO_2_ using nanoporous materials. The reduction of CO_2_ using nanocatalysts is carried out using electrocatalytic, photocatalytic, and heterogeneous thermal catalysis,^60^ in decreasing order of their contribution. The electrocatalytic reduction of CO_2_ has been reported using transition metals,^61^ particularly copper^62^ and tin, noble metals, post-transition metals,^63^ and carbon nanostructures. Thermal catalytic reduction of CO_2_ is carried out using supported noble^64^ and transition-metal nanoparticles.^65^ Nanoporous materials such as metal organic frameworks (MOFs),^59^ porous organic polymers,^66^ covalent organic frameworks (COFs),^67^ carbon nanotubes,^68^ nanoporous carbon,^69^ and nanoporous silica^70^ are used for the capture and storage of CO_2_.^71^

Applications of nanomaterials/nanotechnology in agriculture has grown tremendously, having the second highest growth rate (1.7-fold).^72−74^ Nanomaterials are of interest due to their potential to help address the world’s food security and agricultural challenges such as those caused by pesticides, traditional fertilizers, climate change, irrigation difficulties, and poor soil quality. They are also considered a possible pathway to sustainable fertilizers.^75^ A recent review by Shah et al. discusses how nanotechnology can be applied for soil remediation, fertilizers/pesticides, drought stress, crop growth and seed germination (genetic engineering), water management, and nutrient delivery with a focus on sustainable agriculture.^71,76^

Life science and biomedical applications have driven significant growth in the use of nanoscale materials, as shown in Figure 2 (top). Three notable examples include vaccines, nanozymes, and bioinks with publications growing at a pace ranging between 1.4- and 1.7-fold every year. Vaccines are biological preparations that render immunity to a particular infectious disease by stimulating the immune system to recognize the pathogenic agent. Nanoparticle-based vaccines are an emerging area of research that utilizes nanotechnology to enhance the effectiveness of vaccines.^77−82^ These nanovaccines employ nanoscale materials, such as nanoparticles, liposomes, nanogels, micelles, and dendrimers, as delivery vehicles for antigens and adjuvants, aiming to improve immune responses and vaccine efficacy.^83,84^ Nanovaccines can advance targeted delivery, antigen presentation, stimulation of innate immunity, and a robust T cell response, combined with safety to combat infectious diseases and cancers.^85,86^ Moreover, nanovaccines can be highly valuable in generating effective immunotherapeutic formulations against cancer.^87−92^

Nanozymes are nanomaterials with enzyme-like catalytic activities.^56,93^ These synthetic nanostructures mimic the functions of natural enzymes but offer several advantages, such as better stability, tunable catalytic properties, cost-effectiveness, and easier large-scale production.^94^ Some common types of nanozymes include metal-based nanoparticles such as gold, silver (Ag), platinum; metal oxide nanoparticles such as iron oxide and manganese oxide; and carbon-based nanostructures such as graphene and carbon nanotubes.^95,96^ They have garnered significant interest in various fields, including biomedicine, environmental remediation, and industrial processes.^97^

The use of nanomaterials in bioinks is gaining popularity, especially for formulations used in 3D bioprinting. For instance, using nanomaterials such as clay, graphene, carbon nanotubes, and silica particles can enhance the structural and rheological properties of bioinks and provide bioactive properties such as drug delivery capabilities and antimicrobial effects.^98^ Bioinks containing nanoparticles loaded with a growth factor such as vascular endothelial growth factor (VEGF) or bone morphogenetic protein (BMP) can help to promote angiogenesis and osteogenesis within printed constructs.^99−101^ In addition, bioinks incorporating polymer nanofibrils can be used to build cell culture scaffolds.^102^ Furthermore, bioinks can be functionalized by incorporating luminescent optical sensor nanoparticles, making them suitable for imaging cells while they grow.^101^ Nanoparticle-containing bioinks can be used for printing 3D organs. For instance, a gold nanorod-incorporated gelatin methacryloyl (GelMA)-based bioink is developed for printing 3D functional cardiac tissue constructs.^103^ These examples highlight vast and exciting applications of nanoparticles containing bioinks in 3D printing.^98^

**Figure 3:**
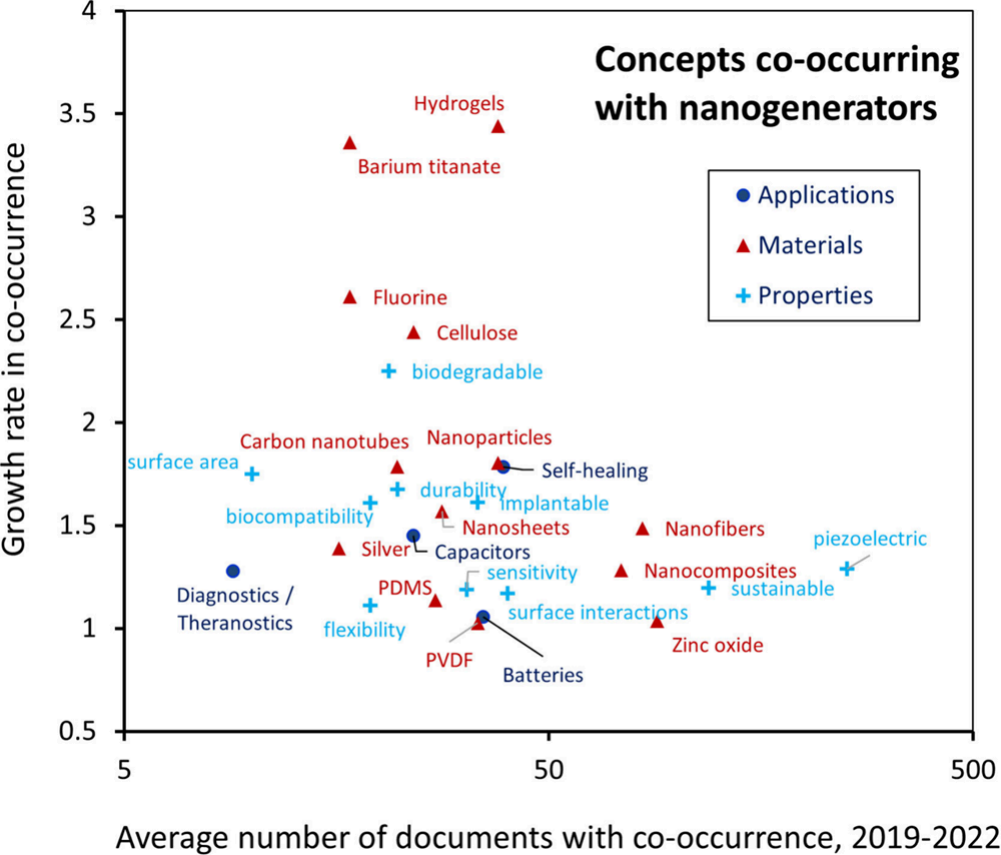
Average 2019–2022 growth rate versus number of publications over that time period for terms co-occurring with nanogenerator applications.

There are several emerging applications which are notable for their relative growth rate and overall number of publications in which they appear from 2019 to 2022. Nanogenerators are an example of such an application, specifically, triboelectric and piezoelectric nanogenerators. These devices generate electrical energy from motion, through charge separation that takes place when two surfaces interact (tribo) or through deformation (piezo).^104^ Their growing frequency in publications appears to be driven by their use to power wearable devices such as human motion sensors^105,106^ and in human–machine interfaces.^107^ We can further understand the context in which the term “nanogenerator” is used in publications by plotting the growth rate and absolute number of co-occurrences of this term with other concepts in the Trendscape maps in our document set. This is shown in Figure 3, where co-occurring terms are classified as material-, application-, or property-related.

Based on this analysis, the emerging materials most prominently associated with nanogenerators are nanofibers^108^ and zinc oxide,^109^ with hydrogels growing particularly quickly. Nanofibers are of interest to triboelectric nanogenerators because of their high surface area (improving the macroscopic charge density), flexibility, and the possibility of synthesizing customized nanofiber materials using electrospinning.^110^ Polyvinylidene fluoride (PVDF) nanofibers have the additional advantage of a strong electrical dipole due to the presence of fluorine.^111^ One-dimensional (1D) ZnO nanomaterials are used in both types of nanogenerators for their piezoelectric and mechanical properties.^112^

## Materials

**Figure 4:**
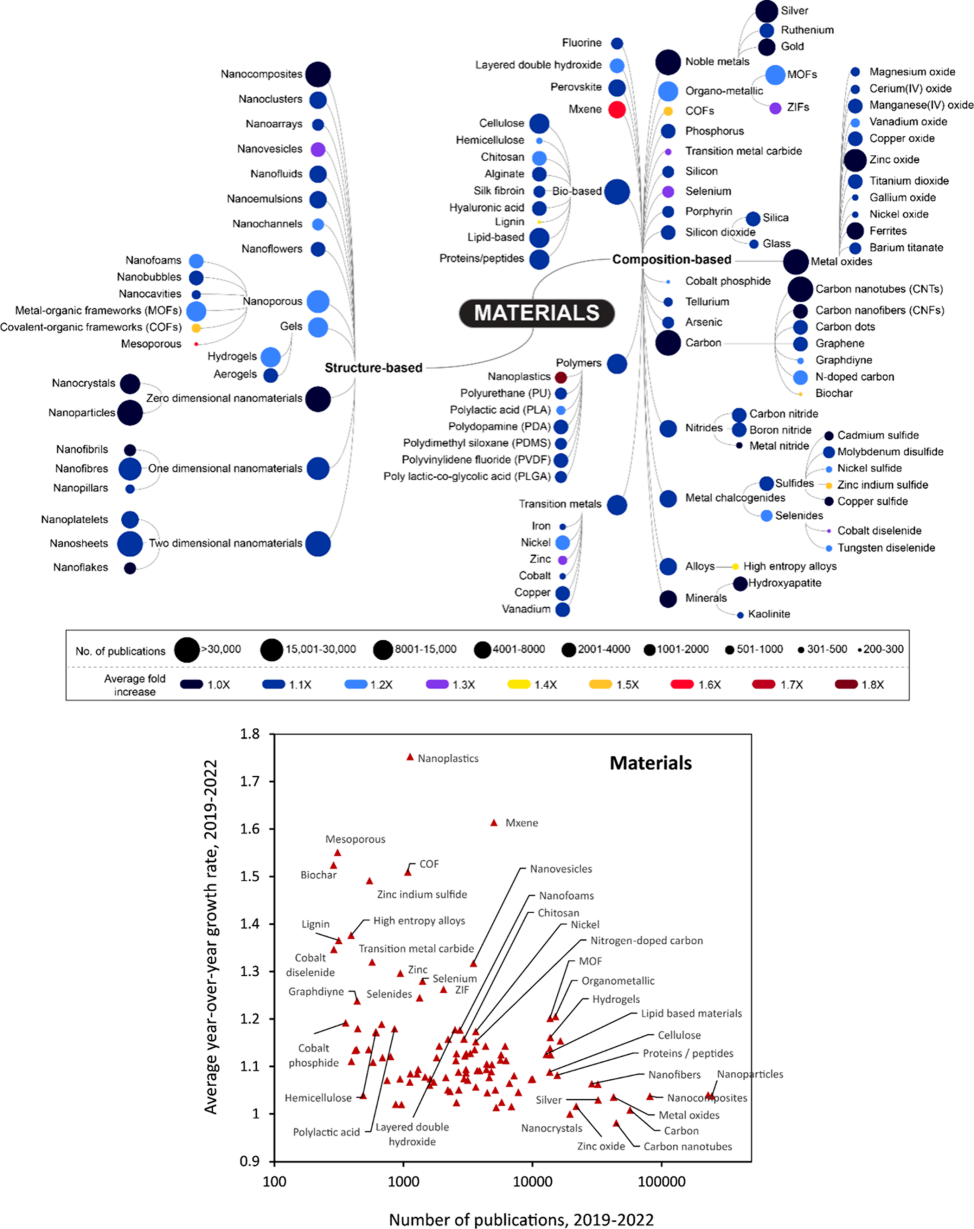
(Top) Conceptual Trendscape map and (bottom) average 2019–2022 growth rate vs absolute number of publications that reference nanoscale materials.

The material-specific Trendscape shown in Figure 4 (top) covers prominently emerging material-related terms that appear in publications involving nanoscale materials.

The terms in the material-specific Trendscape map have been divided into two broad categories: structure-based and composition-based. The former concerns the morphology of nanoscale materials while the latter consists of either nanoscale materials or materials often used in conjunction with nanoscale materials or used to make them. In this section, we briefly discuss materials growing at a moderate to rapid pace (>1.4 fold over the last three years)—nanoplastics, MXenes, COFs, and zinc indium sulfide.

As can be seen in Figure 4 (bottom), the term “nanoplastics” classified under the polymers subbranch of the composition-based materials category has been growing rapidly (nearly doubling) in use since 2019. Nanoplastics are synthetic or modified natural polymers typically defined as being 1 μm or less in size, though some define them as being between 1 and 100 nm.^113,114^ These plastics have three ways of coming into being: intentionally produced for diverse applications, generation during the manufacturing of polymers, or via the fragmentation of larger plastics.^115−117^ Nanoplastics-related terms co-occur with topics such as toxicity,^118,119^ antioxidants, and characterization techniques such as X-ray diffraction.^120−129^ These associations are due mostly to concerns about the effects of nanoplastic waste in the environment. Co-occurrence with nanoparticles is also present due to some nanoplastics being identified as nanoparticles,^130−132^ but publications also discuss other associated nanoparticles (for example, silver and TiO_2_) regarding their removal alongside nanoplastics,^120^ their combined toxic effects,^124^ the identification of nanoplastics in nanoparticle mixtures,^133^ their use in enhancing sensors to quantify nanoplastics,^134^ and for other reasons.

**Figure 5:**
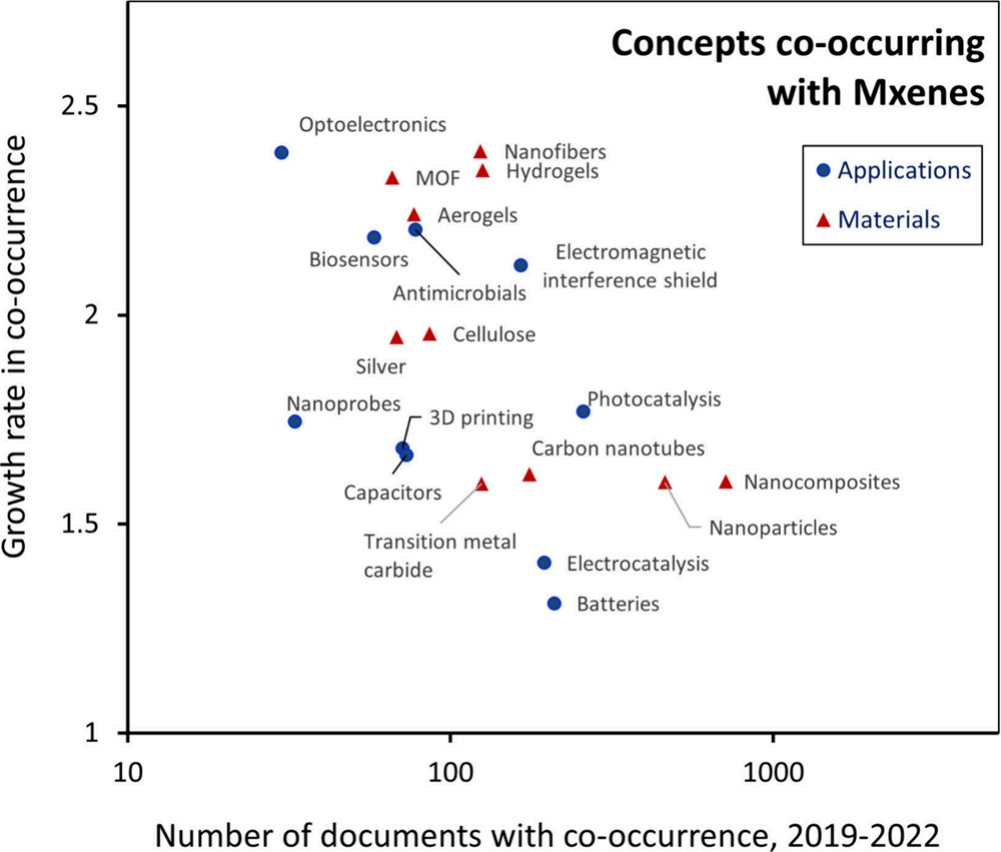
Average 2019–2022 growth rate versus number of publications over that time period for terms co-occurring with MXenes.

MXenes are a class of inorganic 2D materials that have been the subject of growing research interest since they were first reported in 2011.^135^ Prominent applications of MXenes currently are in electrocatalysis,^136−138^ photocatalysis,^139,140^ and batteries.^141,142^ They are well suited for use in these areas due to their high surface area, electrical conductivity, and high degree of versatility through altering their surface functionality and/or combining them with other nanoscale materials. Antimicrobial applications represent an especially fast-growing area of use for the specific MXene Ti_3_C_2_T_*x*_.^143,144^ MXenes are also frequently combined with other nanoscale materials, such as carbon nanotubes, to fully leverage their unique properties. A more complete set of applications and materials that frequently co-occur in documents with MXenes are shown in Figure 5. These trends show that the use of MXenes in applications such as optoelectronics, electromagnetic shields, antimicrobials, and biosensors is growing at a faster rate. Though the number of publications reporting the use of MXenes in batteries and electrocatalysts is high, the growth rate of their co-occurrence is less. Photocatalysis is one application of MXenes in which both the number of publications and the growth rate are high.

Covalent organic frameworks (COFs) are 2D or 3D porous polymeric networks made of one or more covalently bonded monomers. COFs can be designed to be stable and insoluble under a variety of conditions. Furthermore, the chemical or catalytic properties and pore size of COFs can be customized by choosing the appropriate monomer(s).^145,146^ The pore sizes of COFs are in the microporous as well as mesoporous range and are usually less than 10 nm. COFs are interesting for heterogeneous catalysis, which was usually dominated by inorganic materials, as they bridge the gap with homogeneous catalysis due to their level of customizability, which has historically been possible only in homogeneous catalysis.^147^ COFs can act as catalysts in various ways, such as by using functional groups in their covalently bonded networks, as photocatalysts due to their semiconducting properties through π–π interactions,^148^ as catalyst nanoparticles^149^ or enzymes anchored on the skeletons of COFs, using active sites on the walls of the pores,^150^ or in shape-selective catalysis depending on the pore dimensions. Highly active single-atom catalysts are obtained by anchoring them using coordination bonds to the heteroatoms in the COFs to achieve high catalytic activity.^151^

Zinc indium sulfide, also called indium zinc sulfide, is a ternary metal chalcogenide with a layered structure. It is a semiconductor with a bandgap of 2.2 eV which has attracted interest recently for photocatalytic and photoelectrochemical applications.^152^ According to data from the CAS Content Collection, nearly 93% of the publications related to nanostructured ZnIn_2_S_4_ reference photocatalytic or photoelectrochemical applications, which include water splitting,^153^ carbon dioxide reduction,^154^ and removal^155^ or degradation^156^ of pollutants in aqueous media. The major advantages of ZnIn_2_S_4_ include its bandgap, which is suitable for sunlight absorption, band positions aligned to carry out the water splitting reactions/carbon dioxide reduction, the nontoxic nature of its constituent elements, the relatively moderate cost of its constituent elements, and its stability under photocatalytic water-splitting conditions.^152^ However, the widespread application of ZnIn_2_S_4_ is hindered by the high recombination of the photogenerated charge carriers and its limited absorption in the visible region only up to 563 nm.^157,158^ According to the CAS Content Collection data, the most favored nanostructure for ZnIn_2_S_4_ is the nanosheet due to its layered structure. The other nanostructures in which ZnIn_2_S_4_ is reported include the following in descending order of their number of publications: nanocomposites, nanoparticles, microspheres, nanorods, and quantum dots.

Notably, two naturally derived materials appear in Figure 4. The first is lignin, a complex organic polymer found in plant cell walls, which is essential for providing structural support and rigidity. Its abundance and biodegradability make it an attractive material for nanoscience-related applications. For example, lignin nanoparticles can serve as drug delivery carriers by encapsulating pharmaceutical ingredients and can be functionalized with various targeting ligands to enhance specificity and efficacy in drug delivery.^159,160^ Lignin nanoparticles can be incorporated into polymer matrixes to create nanocomposites which can be used in packaging and automotive applications and can also be used for environmental remediation applications including soil remediation, wastewater treatment, and sustainable agriculture.^161,162^ Lignin can be processed into nanofibers which can be used in developing filtration systems and tissue culture scaffolds due to high mechanical strength, high surface area, and biocompatibility.^163−165^ Lignin can also be used to develop nanomaterials which can be used in energy storage applications and catalysis.^161,162,166,167^

The second naturally derived material is biochar, which is made through the pyrolysis of biomass. Its most prominent application is in the removal of pollutants from water, including heavy metals^168^ and organics.^169^ The adsorptive capacity of biochar can be enhanced by modifying it with metals or other chemical functionality. This approach can also be used to impart to it the ability to oxidize organic contaminants using Fenton-like processes.^170^

Extracellular vesicles, which represent a route of intercellular communication and are involved in essential physiological processes, have emerged as powerful tools in various fields including drug delivery, diagnostics, and biotechnology.^171−173^ However, their limited targeting ability, insufficient production yield, and low drug encapsulation capability have hampered their clinical development. Therefore, engineering multifunctional hybrid nanovesicles mimicking natural extracellular vesicles but with favorable adaptability and flexibility has become a key challenge in expanding their application.^174−176^ Such nanovesicles are nanosized vesicles composed of lipid bilayers and/or other materials. They have garnered significant interest due to their unique properties and ability to encapsulate and deliver therapeutic agents, biomolecules, and imaging agents, thus offering opportunities for targeted drug delivery, diagnostics, and therapeutic interventions. Nanovesicles can be composed of lipids, polymers, proteins, or a combination of these materials.^177−181^ They can be loaded with imaging contrast agents or fluorescent dyes for noninvasive imaging of tissues and cells *in vivo*. Nanovesicles derived from stem cells or other cell types hold promise for tissue regeneration and repair by delivering bioactive molecules and promoting cellular signaling pathways.^182−184^

## Properties

**Figure 6:**
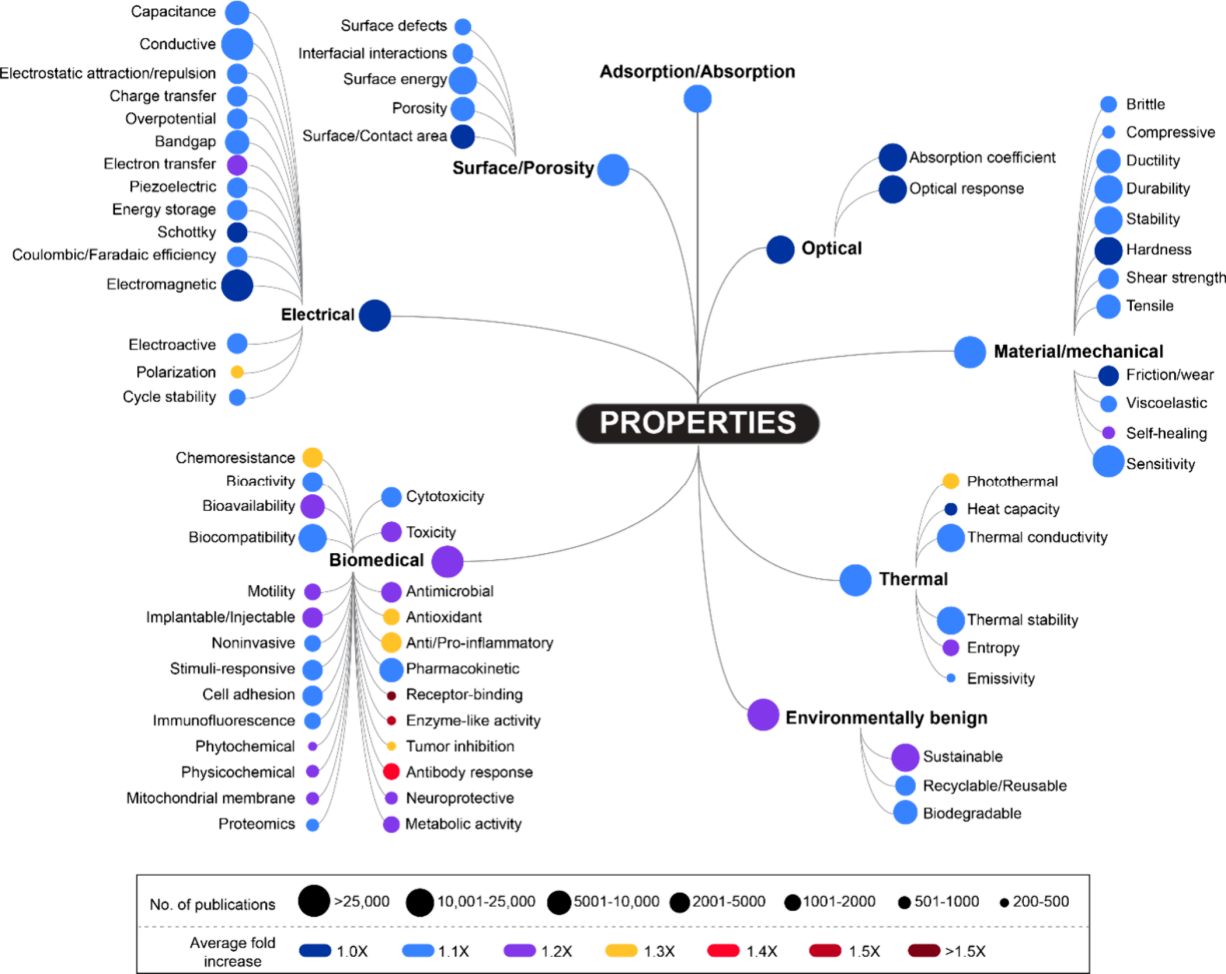
Conceptual Trendscape map from 2019 to 2022 that references nanoscale properties.

The data shown in Figure 6 refer to the use of prominently emerging property-related terms that appear in publications involving nanoscale materials. These property-related terms may be related to the nanoscale materials themselves or to materials that are often combined with nanoscale materials or are used to make them. While the electrical, optical, material/mechanical, thermal, biomedical, and environmentally benign properties all have comparable numbers of publications, the biomedical and environmentally benign ones exhibit the fastest growth.^185^

## Connections between Concepts

To understand connections between concepts in the Trendscape map, we have performed an NLP-based analysis which counts the number of co-occurrences of individual concepts in the same sentences of journal abstracts. This allows us to quantify the degree of connection between any two concepts shown in the Trendscape maps in Figures 2 and 4.

**Figure 7:**
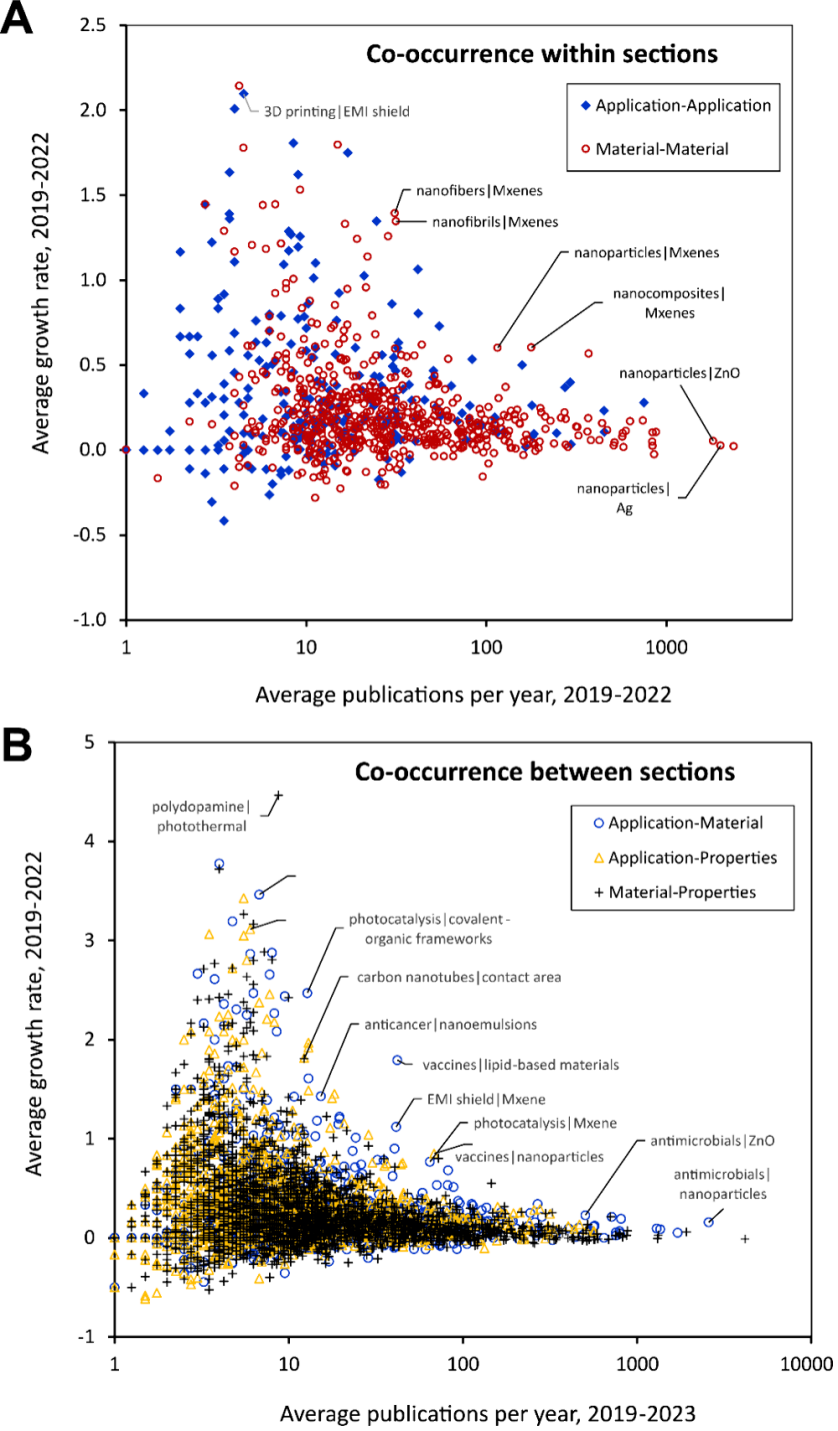
Average year-over-year growth rate versus absolute number of publications from 2019 to 2022 for concepts co-occurring in the same sentence in journal abstracts for concepts (A) in the same Trendscape map and (B) in different Trendscape maps.

The plots in Figure 7 show the average number of documents published between 2019 and 2022 where pairs of terms co-occur in the same sentence (*x* axis) and the average growth rate of documents is presented with those co-occurrences over the same time period (*y* axis). For clarity, combinations are separated into two figures, showing the co-occurrence of concepts within the same maps/broader category (i.e., terms which appear in the application map or in the materials map) and the co-occurrence of terms in different maps/across different broader categories. The general trend observed in this data is that there is a wide range of growth rates for the combinations with relatively low publication frequency (less than 20–30 documents per year), with a long tail extending to high publication numbers but relatively low growth rates.

The most interesting pairs on the graphs, represented by labeled data points, fall into two categories. The first are pairs that have a relatively high growth rate compared with other concept pairs with a similar number of total documents, which represent emerging connections between concepts. The second includes pairs with a fairly high number of documents but a low growth rate, which can be described as well-established or more mature combinations.

Discussed below are a few key observations based on the data shown in Figure 7A. ZnO and Ag, which both co-occur in the same sentence as nanoparticle terms in over 2000 documents between 2019 and 2022, appear to be very well established nanoparticle materials. In contrast, the combination of MXenes with nanofibers and nanoparticles represents a faster growing area of research but with fewer overall publications. Within these combinations, publications where MXenes are combined with nanoparticles are more frequently studied compared with their combination with nanofibers, which is growing more quickly but with fewer overall publications.

In Figure 7B, we see that the combination of vaccines and lipid-based materials appears prominently, with an exceptionally high growth rate given the total number of references for this combination; this can likely be attributed to the large number of publications related to COVID-19 vaccines published in 2021 and 2022. Other biomedical applications (anticancer–nanoemulsions, vaccines–nanoparticles, and antimicrobial–nanomaterials) also appear prominently in this analysis, along with energy conversion or catalysis-related applications (photocatalysis–MXene and photocatalysis–covalent organic frameworks) and electronics applications (EMI shield–MXene).^186,187^

## Applications of Artificial Intelligence in Prominent Nanotechnology

**Figure 8:**
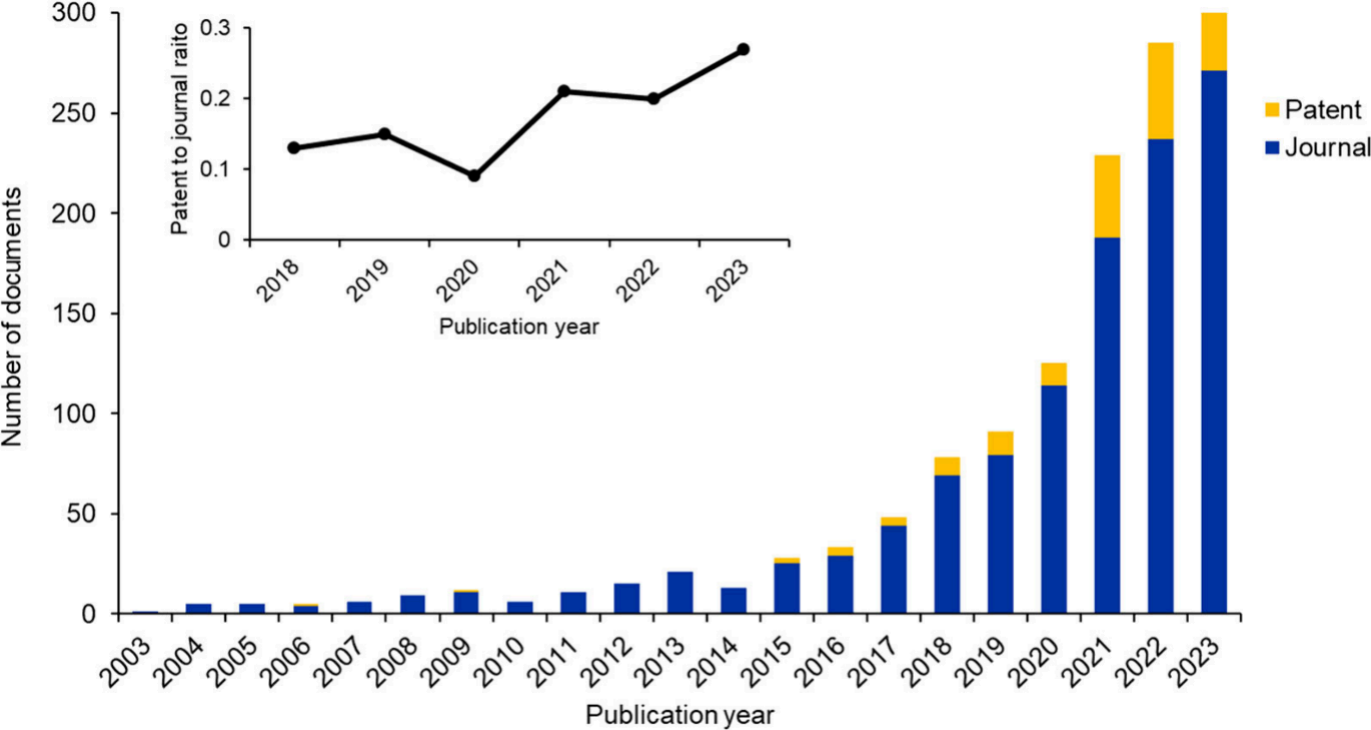
Number of journal and patent publications published per year from the CAS Content Collection that are related to the use of AI in nanoscience-related research areas over the last two decades (2003–2022). The inset shows the trend for the patent-to-journal ratio for the last five years (2018–2023).

Artificial intelligence (AI) can play a significant role in nanorelated research by helping researchers discover novel nanomaterials with desired features, predicting properties and applications of existing and new nanomaterials, and reducing the time required to analyze output data from nanomachines. Our analysis of AI-related publications in the nanoscience/nanotechnology realm in the CAS Content Collection shows that there have been roughly 1600 scientific publications since 2003 (including journal articles, patents, and conference publications) related to the use of AI in nanoscience-associated research. Figure 8 shows the yearly distribution of these publications. Overall, the number of publications has steadily increased in the last two decades, indicating continuous growth in research, development, and commercialization efforts being made in this field. Perhaps unsurprisingly, journal publications dominate the field, with their total number being ∼7 times higher than patent publications, indicative of the nascency of the field. However, the overall patent-to-journal ratio has shown a steady increase in the last five years, indicating the onset and progression toward commercialization of research in this field.

**Figure 9:**
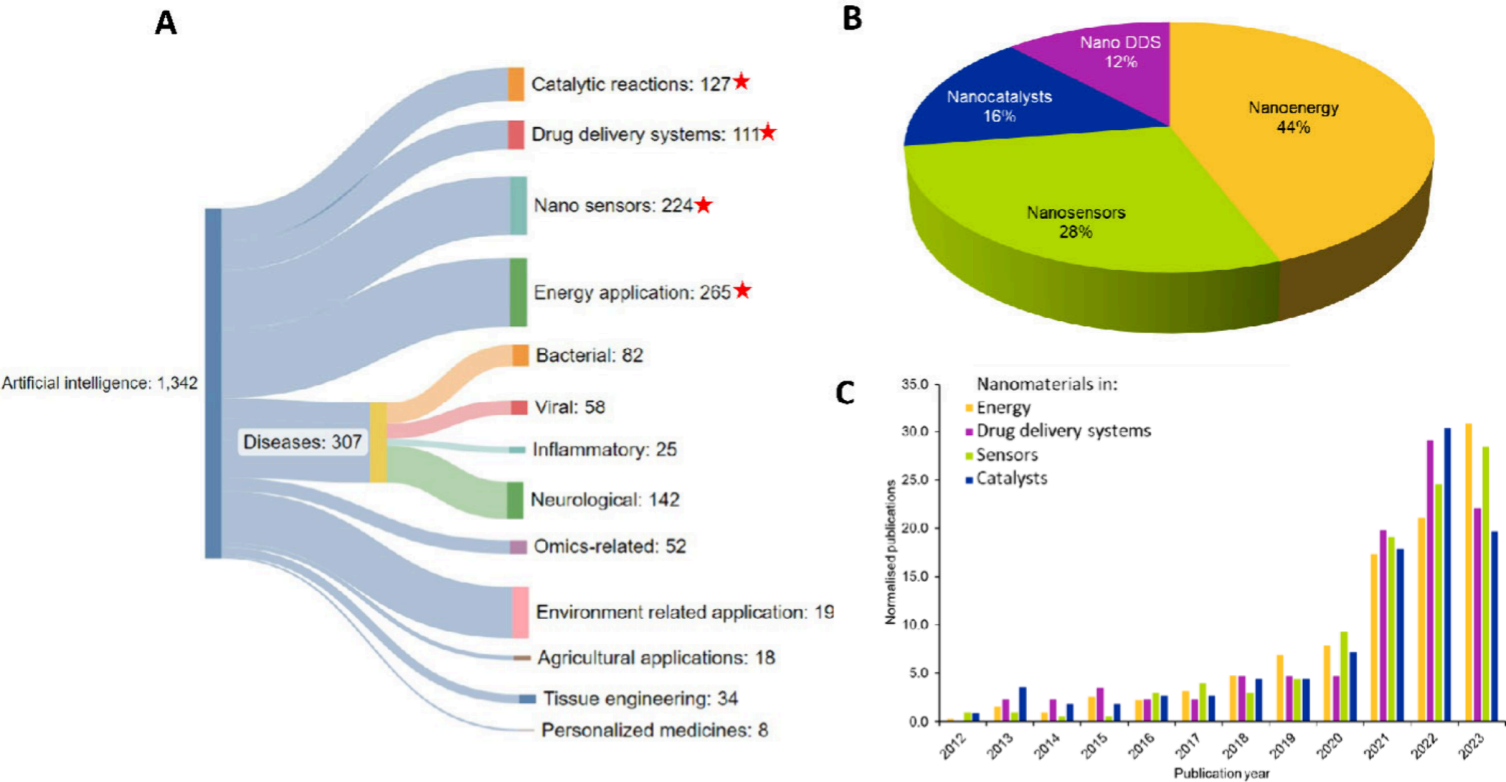
(A) Sankey diagram showing correlations between uses of artificial intelligence with applications in several nanorelated fields derived from the CAS Content Collection. (Branches marked with a red asterisk are explored in panels B and C.) (B) Percentage distribution of AI use in prominent nanorelated fields. (Note: Nano DDS is used for nano drug delivery systems.) (C) Yearly growth for the use of AI in prominent nanorelated fields from 2003 to 2023.

Figure 9A shows a Sankey diagram illustrating the correlation of AI and its different applications in the field of nanorelated research where we see that sensors, energy, catalysis, and drug delivery are prominent areas that show maximum co-occurrence with the use of AI.^188^ The use of AI also shows a high correlation with other applications such as environmental, agricultural, and tissue engineering-related applications and disease areas where AI is being used for detection, diagnosis, and treatment. Among these four prominent fields—sensors, energy, catalysis, and drug delivery—the use of nanomaterials in energy and sensors is the highest as demonstrated by high percentages (44 and 28%, respectively) of scientific publications in these areas (Figure 9B). Further investigation into the prominent fields of nanoscience reveals that the use of artificial intelligence has increased in publications related to the application of nanomaterials in energy and sensors (Figure 9C). A steady increase in the number of scientific publications in the past decade can be seen in relation to these applications, indicating the rising interest of the scientific community in this field. Interestingly, the number of publications discussing the involvement of AI in the other two prominent nanorelated areas—catalysts and drug delivery systems—also shows a steady increase until 2022.

## AI Applications in Nanoscience and Nanotechnology

AI has revolutionized various scientific fields; additionally, advancements in computational approaches and nanomaterials have helped to synergize these fields for various applications across diverse domains. The role of machine learning, where it accelerates the various stages of nanomaterials research such as design, synthesis, characterization, and application, is on the rise.^189^ For instance, advanced sensors such as image, vision, and wearable sensors use AI-based algorithms such as machine learning and neural networks to analyze complex and multidimensional data output generated by them^190,191^ AI-enabled nanosensors monitor data in real time, improving the ability of healthcare providers to detect diseases, track disease onset and development, and continuously monitor health conditions.^192−194^ For drug delivery using nanoparticles, AI can help optimize various aspects of drug delivery such as drug design, optimizing drug formulation, controlled drug release, enhancing localized drug delivery, target penetration, etc.^195−197^ The use of AI-enabled sensing technologies can help in designing nanoparticle-based personalized medicine and treatment optimization in the future with real-time monitoring and feedback capabilities. AI-enhanced nanogenerators such as piezoelectric nanogenerators (PENG) and triboelectric nanogenerators (TENG) can bring about paradigm shifts to the sector of energy harvesting^192,198^ By using AI algorithms, these nanogenerators can optimize the energy-harvesting efficiency. AI-enhanced nanogenerators can use predictive analytics to efficiently manage energy generation. AI can also benefit the field of nanocatalysts, where AI-enabled algorithms analyze vast data sets of nanomaterials to predict catalyst reactivity, performance, and reaction mechanisms.^199,200^ AI methodologies such as machine learning are enabling the design of high-performance composites by accurately predicting their properties.^201^ In addition, AI-based programs can explore chemical repositories to identify novel nanomaterial compositions and combinations with unprecedented catalytic activity. In conclusion, AI has the potential to significantly accelerate nanoscience and nanomaterial development.

Overall, leveraging AI in the area of nanoscience is expected to improve the efficiency, precision, and scalability of its applications.^202^ The use of AI in nanoscience is expected to play a pivotal role in solving some of the critical problems in health care, energy, and environmental sustainability. Interdisciplinary collaboration between AI experts and scientists working in nanoscience will pave the way for achieving the full potential these two promising research areas.^203^

## Environmental and Health Concerns Related to Nanoscience

The development of novel technologies raises concerns related to their impact on the environment and on humans. In this regard, compliance with the “One Health” concept is considered to be an important requirement for novel technologies. “One Health” aims to balance the benefits and impacts of various processes on the health of people, animals, and ecosystems.^204^ It is important for applications based on nanoscience to be compliant with the “One Health” concept to balance its benefits and its associated risks. Some of the concerns raised regarding nanoscience are associated with the use of high amounts of harmful chemicals during their synthesis and the impact of nanomaterials on human health.

The synthesis of nanoparticles involves the use of high amounts of harmful capping agents, reducing agents and solvents, raising concerns about the health and environmental impact of such synthesis methods.^205^ In an effort to resolve this issue, alternative greener and less harmful capping and reducing agents based on polysaccharides, small molecules, and biomolecules are being studied for nanomaterial synthesis.^206^ Plant-based materials are proposed as alternative sources for the green synthesis of nanomaterials.^207−209^ Biosynthesis, where microbes are used for carrying out the reduction process, has been recognized as an alternative route for the green synthesis of nanomaterials.^210,211,208^

Due to their small size, nanomaterials can enter biological systems, posing health risks. Concerns have been raised about such risks.^212^ It is becoming increasingly important to address these concerns as various applications of nanoscience in agriculture and food are getting reported. These concerns need not hinder research in nanoscience as it has been enabling many sustainable energy and environmental remediation applications and the benefits outweigh the potential risks.^213^ Conducting human and environmental toxicology studies of the nanomaterials and communication between the various stakeholders will help address these concerns.^212^

## Summary, Outlook, and Further Opportunities

The field of nanoscience and nanotechnology is poised for unprecedented growth, driven by advancements in fundamental research and technological innovations. The development of novel materials, the exploration of unique properties, and the expansion of applications are driving significant opportunities for future research and technological growth. In this review, we mapped the emerging topics in nanoscience and nanotechnology with regard to applications, materials, and properties by using natural language processing (NLP) analysis of the nanoscience-related publications since 2019. NLP offers a transformative potential by accelerating the discovery of new materials and optimizing existing ones. Through the analysis of vast amounts of scientific literature and patents, NLP algorithms can identify patterns, trends, and novel materials with the desired characteristics. This approach reduces the time and resources needed for experimental trials, enabling faster innovation cycles.

Our analysis showed that the applications with the highest growth are from areas including biomedical, energy, environmental, food, and technology.^214^ Drug delivery, catalysis, energy, and sensors were identified as the applications with the highest numbers of publications. The development of new nanomaterials with tailored properties is an exciting frontier. A wide array of materials are being explored, including graphene, carbon nanotubes, and quantum dots, which exhibit unique electrical, mechanical, and optical characteristics. These materials have the potential to address critical challenges in energy storage, environmental protection, and sustainable manufacturing. Nanoscale catalysts can enhance the efficiency of chemical reactions, leading to greener industrial processes. Nanomaterials can contribute to the development of high-capacity batteries and supercapacitors, which are essential for the transition to renewable energy sources. Materials which have multidisciplinary applications such as MXenes, covalent organic frameworks, and zeolitic imidazolate frameworks as well as those with specific applications such as zinc indium sulfide and high-entropy alloys exhibit considerable growth. In general, nanomaterials with properties suitable for energy and environmental applications are those that grew the most. The noteworthy emerging properties are related to biomedical applications, sustainability, and energy conversion. Co-occurrence analysis between the various emerging topics provided deeper insights linking application, material, and property topics. An analysis of the use of artificial intelligence (AI) in nanoscience showed its growth trends and its presence across broader and specific applications.

While this review discusses the impressive capabilities of nanoscience, there are also wide opportunities for growth and improvement, including efforts in developing materials with customized properties for specific applications, such as smart sensors, advanced coatings, and high-performance composites, and focusing on ecofriendly and recyclable nanomaterials to reduce environmental impact and promote sustainability in manufacturing and product life cycles. The morphologies and sizes of nanoscale objects are determinants of their functionality, but in many cases, the morphology of nanostructures depends strongly on the materials used. For example, although gold and carbon can be fashioned into a broad range of nanostructures, other materials are significantly more limited in the structures they can assume. The ability to control nanostructures for a given material would allow the replacement of expensive materials with less expensive ones or the incorporation of the functions of one material into another. While the atomic structure and composition of a material may determine the structures it can form, templating or stabilizing components may allow nominally unstable structures to be obtained for a given material. A more readily attainable goal would be to control the structures of carbon nanotubes (and potentially carbon nanostructures, in general). While carbon nanotubes are readily available, single-walled and multiwalled nanotubes require either high temperatures, corrosive reagents, or difficult separation and purification steps^215^ and thus high costs. The ability to generate conducting or semiconducting carbon nanotubes would make a variety of applications more attainable. Carbon nanotubes may be formed in a variety of sizes and stereochemistries, many of which are not currently available but which would likely be useful in sensors. The ability to generate morphologies less dependent on the source material would likely expand the scope of nanomaterial use. The synthesis and applications of nanomaterials are multidisciplinary efforts due to the diversity of their applications.^216−218^ Hence, it is important to foster collaborations among scientists from various disciplines to reap the benefits offered by these materials. The multidisciplinary nature of nanoscience research also poses challenges in patenting discoveries such as difficulty in proving the novelty, the need for multidisciplinary panels, and the lack of standardized terminology.^219^

The future of nanoscience and nanotechnologies is rich with potential driven by the continuous exploration of new materials, innovative applications, and the integration of cutting-edge technologies. By focusing on the development of tailored materials, advancing applications in key sectors, and harnessing the power of novel approaches, including computational linguistics, machine learning, and deep learning models, to process human language, we can unlock new opportunities for growth and make significant strides toward addressing global challenges. As we move forward, interdisciplinary collaboration, sustainable practices, and strategic investments will be crucial to realizing the full potential of nanotechnology and driving meaningful progress in the field.

## Supporting Information Available

Methods; NLP analysis; co-occurrence analysis; and search strings (PDF)
