# Supplementary material for: Nanoscience in Action: Unveiling Emerging Trends in Materials and Applications
Source: ACS Omega. 2025 Feb 17;10(8):7530–48. doi: 10.1021/acsomega.4c10929 (PMC11886759; doi:10.1021/acsomega.4c10929)
Supplement: Supplementary file 1 — ao4c10929_si_001.pdf [file ao4c10929_si_001.pdf]

Supporting Information for

Nanoscience in Action: Unveiling Emerging Trends in Materials and Applications

Kevin J. Hughes†[1], Magesh Ganesan†[2], Rumiana Tenchov†[1], Kavita A. Iyer†[1], Krittika Ralhan†[2], Leilani M Lotti Diaz†[1], Robert E. Bird†[1], Julian M. Ivanov[1], Qiongqiong Angela Zhou*[1]

[1]CAS, a division of the American Chemical Society, Columbus, OH, US 43210, USA

[2]ACS International India Private Limited, Pune, Maharashtra, 411044, India

†These authors contributed equally to this paper

*Corresponding author: qzhou@cas.org

Methods

NLP analysis

1. Data extraction

Using a custom-made search query ("?nano? AND (journal/dt) OR (patent/dt)")), we identified around 3 million documents related to nanoscience and nanotechnology in the CAS Content Collection. The documents include journal articles, patents, conference proceedings, and preprints published from the year 2003 onwards. We then used a novel Natural Language Processing (NLP) to identify the emerging topics within these documents. For identifying candidate phrases, Natural Language Toolkit (NLTK) procedures lemmatization and removal of English stop words by using the python library implementation of NLTK were performed on all n-grams containing 1 to 6 words from the abstract and title of the documents. In addition, we only considered phrases that were found in at least 100 documents.[3]

2. Data processing

After applying the NLTK procedures mentioned above we obtain our final set of candidate phrases. For each of the candidate phrases we build its corresponding Citation Graph (CG). A CG is a directed graph where the nodes are documents, and the edges are citations of documents (Figure S1).

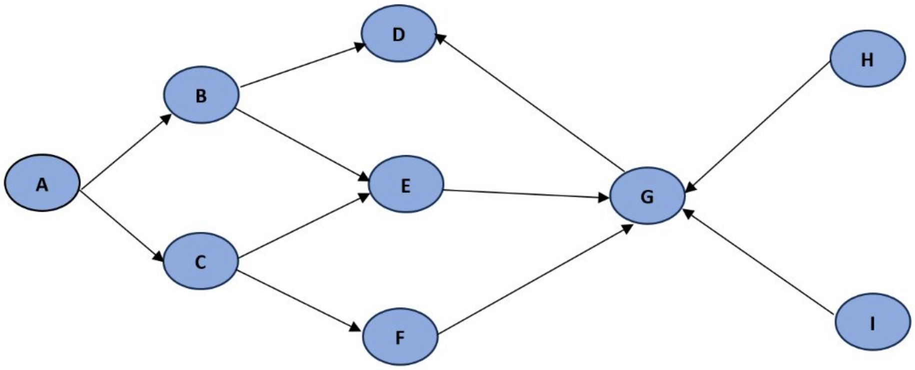

Figure S1. An example of a Citation Graph showing connections between nodes.

a. Scientific concept measures

To evaluate candidate phrases and identify them as scientific concepts we considered the measures derived from the CG (Figure S1).

A good measure to gauge scientific concepts would be one that evaluates the cohesiveness of the CG. Cohesion, in a scientific context, refers to the state of cohering or sticking together of alike entities. There are many ways to measure the graph cohesiveness in respect of different graph attributes. For our purposes we use the Beta index which measures the level of connectivity in a graph and is expressed by the relationships between number of edges over the numbers of nodes (Eq. 1).

$$\beta_{\text{index}} = \frac{E}{V} \qquad \text{Equation 1.}$$

where E = number of edges (citations)
V = number of nodes (documents)

b. Emerging topics measures - Rate of publications and citations

To evaluate the emergence of scientific topics we consider the distributions of publications and citations over the years (Figure S2). While publications themselves are regarded as a way to establish a research topic, the number of citations is usually considered as a better measure of the impact the topic has in the scientific community.

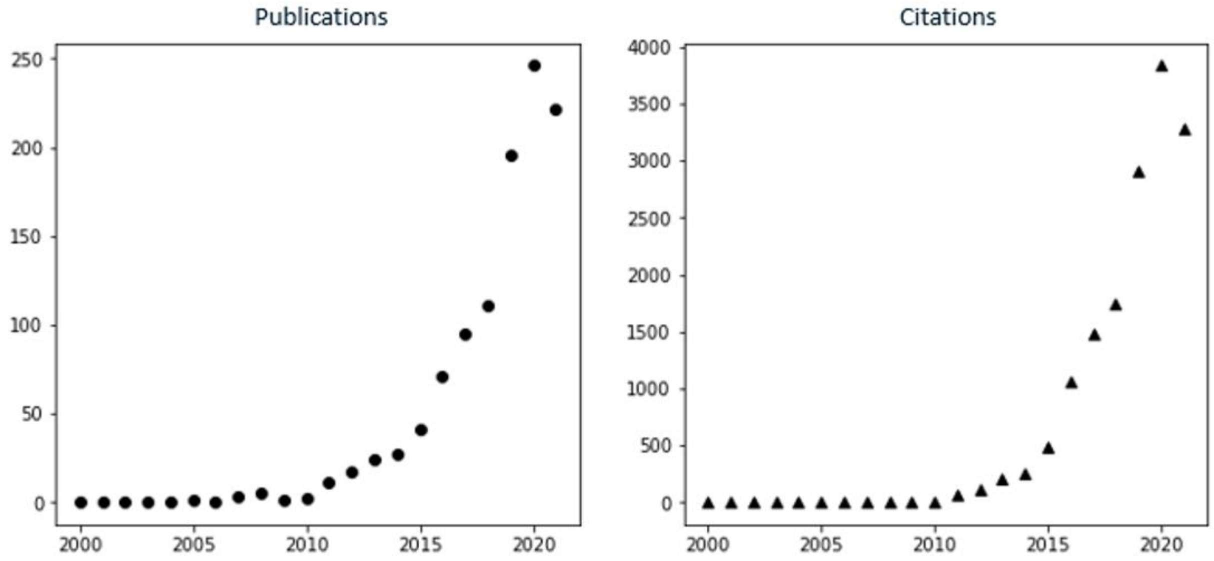

Figure S2. Distributions of publications and citations of an example phrase between the years 2000 and 2022.

A good measure for emergence is the rate of increase/decrease of the publications/citations over a certain time limit. We use the actual yearly data distributions of publications/citations to fit a mathematical function that accurately approximates the observed data. We examined a few distinct functions such as sigmoid, gaussian, exponential, and polynomial with the best approximations obtained using a quartic polynomial function (Figure S3) for the dataset.

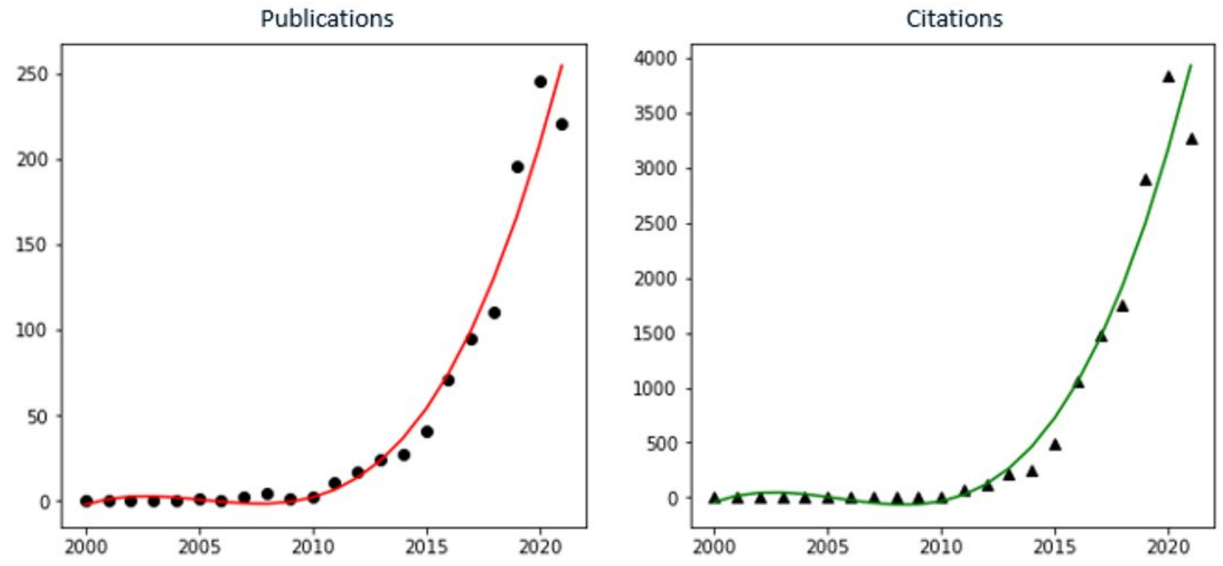

Figure S3. The polynomial function that approximates the actual data of publications, citations of an example phrase between years 2000 and 2022.

Using the derived mathematical function gives us a better solution to the data fluctuations problem and allows us to make predictions and, most importantly, to introduce our measure of emergence. As a measure of emergence, we define the slope of the curve of the mathematical function at the point of. Figure S4 illustrates the measures of emergence for publications and citations of the example phrase at year 2014.

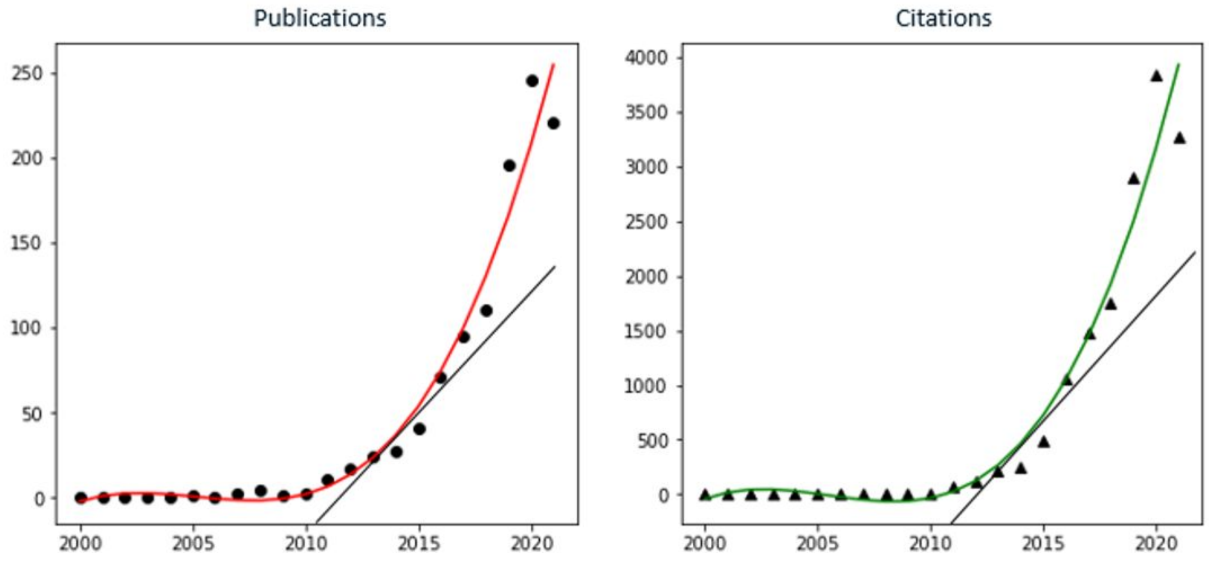

Figure S4. The measures of emergence for publications and citations of an example phrase at the point of interest (year 2014).

c. Predictions validation

As mentioned above, the introduction of a mathematical function to approximate observed data readily allows us to make quick predictions for future publications and/or citations of research topics. To validate the accuracy of the predictions, we fit the polynomial function by using the available bibliographic data for the years 2000 to 2019 and make predictions for publications and citations for years 2020 to 2022 (Figure S5). As can be seen from the results the derived mathematical function produces highly accurate predictions with an average R^2 well above 0.9 for the years 2020 and 2021, and only the R^2 of the prediction for publications in 2022 is below 0.9. This gives us high confidence that the derived function approximates very well with the observed data and therefore can be used successfully to evaluate the emergence of research topics.

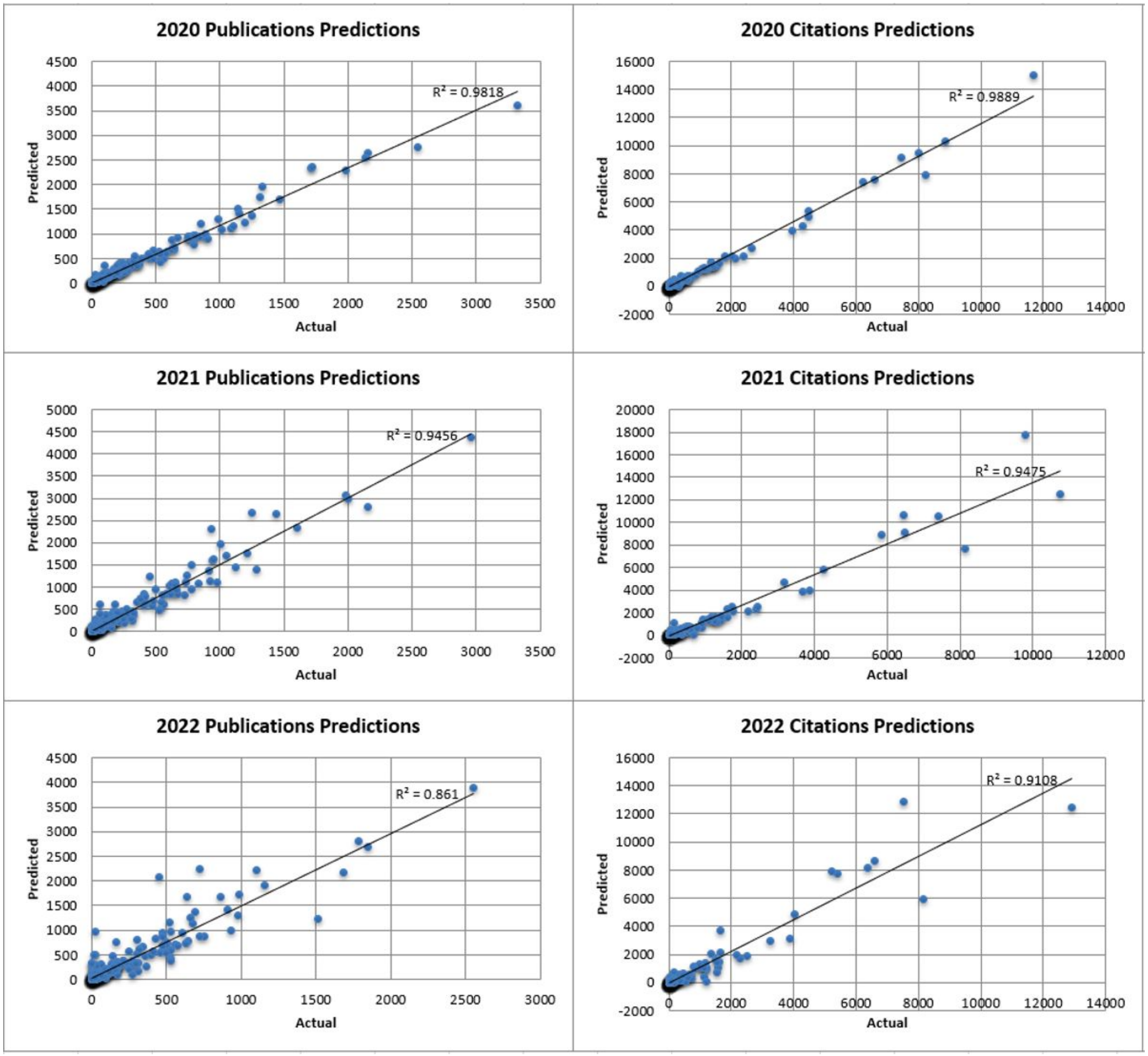

Figure S5. Predictions of publications and citations for the years 2020-2022.

Applying the NLP analysis described above and the >100 documents constraint, 306,353 candidate phrases were identified. For each phrase, the NLP method calculated the publication rates and number of documents in which a particular phrase appeared. Publication rate for a given year is the difference in the number of documents compared to the previous year. We used the top 20,000 phrases with the highest average of the publication rates for the years 2020, 2021 and 2022 for further analysis. From these 20,000 phrases, we manually picked those phrases which can be identified with an application or material or property. For example, phrases such as "play crucial" and "highlight potential" which cannot be connected with any application or material or property are ignored. The selected phrases are then grouped into topics. For example, "solar cells", "photovoltaics", and "perovskite solar" are grouped under 'solar cells'. Then these grouped topics are categorized into applications, materials, and properties.
Out of the various applications which were identified, we selected four applications with the most number of documents for an in-depth analysis.

Co-occurrence analysis

To calculate the rate of co-occurrence (Figures 3, 5, and 7), the same grouped topics that were identified using the NLP analysis described above and shown in the mind maps in Figures 2 and 4 were used. For each pair of topics, the number of document title and abstract in which any phrase in one topic appeared in the same sentence as any phrase in another topic was counted. For example, a document where the term "solar cells", "photovoltaics", or "perovskite solar" appeared in the same abstract sentence as "nanocavities" or "nanocavity" was counted as a co-occurrence of the topics "solar cells" and "nanocavities".

This analysis was conducted for journal and patent documents published between 2019 and 2022. The average rate of increase (y-axis of Figures 3, 5, and 7) was calculated as the average year-over-year change in co-occurring document count over that period.
